# Glycolytic reprogramming impairs chondrocyte function in broilers with femoral head necrosis

**DOI:** 10.1080/01652176.2025.2579940

**Published:** 2025-10-29

**Authors:** Hongfan Ge, Anqi Wang, Yanyan Zhang, Zhenlei Zhou

**Affiliations:** Department of Clinical Sciences, College of Veterinary Medicine, Nanjing Agricultural University, Nanjing, Jiangsu, China

**Keywords:** Glycolysis, oxidative phosphorylation, ECM degradation, metabolic reprogramming, mitochondrial dysfunction, femoral head necrosis, broiler

## Abstract

Cartilage extracellular matrix (ECM) destruction is a hallmark of femoral head necrosis (FHN) in broilers. Chondrocytes undergo metabolic reprogramming under stress to maintain function. However, the metabolic alterations in FHN chondrocytes remain unclear. This study aims to investigate the overall changes of metabolic state in FHN chondrocytes and its functions. Femoral head cartilage of healthy and FHN broilers was collected for non-targeted metabolome and transcriptome analyses. Additionally, primary chondrocytes were isolated from femoral head cartilage of control (CON) and FHN broilers for bioenergetic analysis and mechanistic investigation. Multi-omics profiling revealed significant enrichment of the glycolysis pathway, decreased levels of tricarboxylic acid cycle metabolites (citrate and malate), upregulation of the lactate dehydrogenase A (*Ldha*) gene, and downregulation of genes encoding mitochondrial complexes in cartilage from FHN broilers. Compared with primary chondrocytes isolated from CON broilers, FHN primary chondrocytes exhibited elevated basal extracellular acidification rate (ECAR) and increased lactate production. Concurrently, the basal respiration of FHN chondrocytes was decreased, accompanied by unbalanced mitochondrial dynamics and decreased ATP production. Furthermore, fructose-1,6-bisphosphate (FBP) or rotenone treatment was used to mimic the metabolic shift from oxidative phosphorylation to glycolysis, resulting in downregulation of matrix synthesis genes and upregulation of matrix degradation genes in CON primary chondrocytes. Glycolysis inhibition suppressed matrix degradation gene expression in FHN chondrocytes. These findings suggest that glycolytic reprogramming occurs in FHN chondrocytes, and targeting glycolysis may alleviate ECM destruction in FHN broilers, providing a novel insight into the pathological mechanisms of FHN.

## Introduction

Chicken meat ranks as the second most consumed source of animal protein worldwide, owing to its favorable nutritional profile and affordability (Xu and Yin 2024). In response to the growing demand for consumption, rapid body weight gain has been established as a core breeding objective in modern broilers. Particularly under intensive management, fast growth increases the risk of leg disorders in broilers (Liu et al. 2023). Femoral head necrosis (FHN) is a critical leg issue, leading to significant animal welfare concerns and production loss (Hul et al. 2021). Accumulating evidence indicates that cartilage extracellular matrix (ECM) destruction is a key pathological feature during the progression of FHN in broilers (Ge et al. 2024; Yu et al. 2022). As the predominant cell type in cartilage, chondrocytes maintain ECM homeostasis *via* a balance between synthesis and degradation (Alcaide-Ruggiero et al. 2023; Yu et al. 2020). ECM progressive degradation underlies the ECM destruction of cartilage (Shi et al. 2019). Due to the avascular nature of cartilage, which limits oxygen and nutrient diffusion, chondrocytes rely on a unique metabolic pattern to sustain cell function (Zheng et al. 2021).

Chondrocytes primarily depend on glycolysis for glucose utilization, generating over 75% of total ATP (Ohashi et al. 2021). Despite limited reliance on mitochondrial oxidative phosphorylation (OXPHOS) for energy supply compared with most cell types, the mitochondria-derived reactive oxygen species (ROS) contribute to maintaining redox balance in chondrocytes (Martin et al. 2012). Dysregulation of either metabolic pathway may lead to lactate accumulation, energy deficit, or oxidative stress (Wu et al. 2023), ultimately resulting in cellular metabolic stress. Under stress, chondrocytes attempt to preserve cell function by metabolic reprogramming, characterized by a shift from one metabolic pathway to another (Zheng et al. 2021; Liao et al. 2024). Yu et al. reported that glucocorticoid treatment of chondrocytes upregulated gene expression related to lactate dehydrogenase (LDH) and pyruvate kinase M2 (PKM2), key enzymes in glycolysis (Yu et al. 2024). Given that glucocorticoid induction is the classical method for modeling FHN (Zhang et al. 2019), these findings indirectly indicate an enhancement of glycolysis in FHN chondrocytes. Our previous research revealed mitochondrial abnormalities and oxidative stress in the cartilage of FHN broilers (Ge et al. 2024), suggesting that mitochondrial OXPHOS may be affected in FHN chondrocytes. However, the overall metabolic reprogramming remains undefined in FHN cartilage, and it is unclear whether targeting energy metabolism could alleviate the imbalance between catabolic and anabolic processes in FHN chondrocytes.

Therefore, the objectives of this study are to systematically investigate the metabolic characteristics and bioenergetic alterations in FHN chondrocytes and to further investigate the role of metabolic reprogramming in cellular function *via in vitro* studies. These results will contribute to a deeper understanding of the metabolic alterations in FHN cartilage and offer a new perspective on the pathological mechanisms of FHN.

## Materials and methods

### Sample collection

All procedures involving the collection of biological samples were reviewed and approved by the Animal Protection and Use Committee of Nanjing Agricultural University (#NJAU. No20241104085). 28-day-old male Hubbard Efficiency Plus broilers were obtained from a commercial farm in Jiangsu Province. All chickens had a uniform genetic background and consistent husbandry conditions. Based on the Bristol gait score (GS) scale (Granquist et al. 2019), broilers scoring 3 to 5 were identified as lame broilers. These birds were weighed and then humanely euthanized *via* cervical dislocation. Following previously established protocols (Ge et al. 2024), the proximal femoral articular surface was surgically exposed and assessed using the FHN score standard (Durairaj et al. 2009). Based on the evaluation, broilers were divided into two groups: femoral head separation (FHS) group and femoral head separation with growth plate lacerations (FHSL) group, the latter also referred to as the FHN group. Broilers exhibiting normal gait and no pathological abnormalities were selected as the control (CON) group. Femoral head cartilage samples from 12 broilers per group were collected for histopathological analysis, transmission electron microscopy (TEM), and molecular biology experiments. Femur and tibia samples were collected to assess bone parameters. Furthermore, femoral head cartilage was collected from 6 broilers in each of the FHN and CON groups and stored at −80 °C for metabolomic analysis and RNA sequencing (RNA-seq).

### Histological staining

A 4% paraformaldehyde (PFA, Servicebio, Wuhan, China) solution was used to fix the femoral cartilage samples. Following fixation, decalcification was performed using 10% ethylenediaminetetraacetic acid (EDTA, Solarbio, Beijing, China) at room temperature over a 4-week period. After dehydration and clearing, the cartilage was embedded in paraffin and sectioned continuously along the sagittal plane. Serial tissue sections of 4 μm thickness were generated for Hematoxylin and Eosin (H&E), Toluidine Blue, Alcian Blue, Safranin O/Fast Green, and Periodic Acid-Schiff (PAS) staining. The staining intensity of Safranin O, Alcian blue, and Toluidine blue reflects the proteoglycan content (Moo et al. 2022; Schmitz et al. 2010).

### Western blot analysis

Total protein was extracted from cartilage tissue using radio immunoprecipitation assay lysis buffer (RIPA, Beyotime, Shanghai, China), and detailed protocols are provided in the Additional file 1.

### TEM assay

Cartilage samples were fixed and sectioned as previously described (Ge et al. 2024). Images of multiple fields were acquired at 8000× magnification using a TEM (Hitachi, Tokyo, Japan) to evaluate the morphology and ultrastructure of mitochondria within chondrocytes. The number and size of mitochondria in chondrocytes were quantified using ImageJ software. The aspect ratio was defined as the ratio of the major to the minor axis of mitochondria. Samples from three animals per group were analyzed, and five randomly selected fields per sample were used for statistical analysis.

### Skeletal parameter measurement

All adherent soft tissues were carefully removed from the collected femur and tibia. Morphometric analysis, bone mineral density (BMD), and bone biomechanical testing assessment were performed as described in our previous publication (Ge et al. 2024; Zhang et al. 2024).

### LC-MS/MS analysis for non-targeted metabolome

Femoral head cartilage samples were collected from six broilers in each of the CON and FHN groups for untargeted metabolomic analysis. Approximately 100 mg of tissue per sample was cryogenically ground and extracted with prechilled 70% methanol (Macklin, Shanghai, China). The vortexed mixture was kept on ice for 15 min and then centrifuged at 12,000 rpm for 10 min at 4 °C. The supernatant was transferred and incubated at −20 °C for 30 min. Following a subsequent centrifugation, the final supernatant was obtained for LC-MS/MS analysis. Liquid chromatography (LC) was performed using a reversed-phase column (ACQUITY Premier HSS T3, 2.1 × 100 mm, 1.8 μm, Waters, Massachusetts, USA). The chromatographic separation employed two mobile phases: phase A consisted of 0.1% formic acid in water, and phase B contained 0.1% formic acid in acetonitrile (both from Merck, Darmstadt, Germany). The column temperature was maintained at 40 °C with a flow rate of 0.4 mL/min. The gradient elution program was set as follows: 5% B at 0.0 min, 20% B at 2.0 min, 60% B at 5.0 min, 99% B from 6.0 to 7.5 min, returned to 5% B at 7.6 min, and maintained until 10.0 min. Raw data were subsequently acquired using a Q Exactive HF-X mass spectrometer (Thermo Scientific, Massachusetts, USA) in both positive and negative electrospray ionization (ESI) modes. The main parameters included a spray voltage of 3.5 kV, sheath gas flow rate of 30 arb, auxiliary gas flow rate of 5 arb, ion transfer tube temperature of 320 °C, and vaporizer temperature of 300 °C. Raw data were preprocessed, and metabolites were identified and annotated according to the method described by Li et al. (2025). To analyze differences between the CON and FHN groups, principal component analysis (PCA) and orthogonal partial least squares discriminant analysis (OPLS-DA) were conducted. Differentially expressed metabolites (DEMs) were defined by a variable importance in projection (VIP) > 1 and *p* < 0.05. Pathway enrichment analysis of the DEMs was conducted using the Kyoto Encyclopedia of Genes and Genomes (KEGG) database.

### RNA-seq

Femoral head cartilage samples were collected from three broilers in each of the CON and FHN groups. Total RNA was extracted according to a previously described protocol (Miao et al. 2022), and RNA quality was assessed using the Qsep400 Bioanalyzer (Bioptic, Taiwan, China). Qualified RNA samples were submitted to Metware Biotechnology Co., Ltd. (Wuhan, China) for library construction, quality control, and sequencing on the Illumina NovaSeq X Plus platform (California, USA). Low-quality reads were filtered out using FastQC (Babraham Bioinformatics, Cambridge, UK) to generate clean data, which were then aligned to the reference genome using Hisat2 (Johns Hopkins University, Maryland, USA) (Cui et al. 2024). Gene expression levels were quantified as fragments per kilobase of transcript per million mapped reads (FPKM) using featureCounts (WEHI, Melbourne, Australia), and the gene set enrichment analysis (GSEA) was performed using GSEA software (Broad Institute, USA). Differentially expressed genes (DEGs) were identified using DESeq2 (Bioconductor, Heidelberg, Germany) with thresholds of |${\text{log}}_{2}\text{FC}$| ≥ 0.58 and *p* < 0.05. The sequencing data have been submitted to the Sequence Read Archive (SRA) of the National Center for Biotechnology Information (NCBI) under BioProject accession number PRJNA1211264.

### Primary chondrocyte culture

Femoral head cartilage was collected from 10 broilers in each of the FHN and CON groups. For cell culture, cartilage was cut into approximately 1-mm-thick fragments, washed repeatedly with sterile phosphate-buffered saline (PBS, Servicebio, New York, USA), and immersed in Dulbecco’s Modified Eagle Medium(DMEM, Gibco, Wuhan, China)with 5% Penicillin-streptomycin (Servicebio, Wuhan, China) for 15 min, followed by a final PBS rinse. The cartilage fragments were digested with 0.25% trypsin (Biosharp, Hefei, China) for 30 min, and then chondrocytes were isolated by digestion with 0.1% hyaluronidase (Biosharp, Hefei, China) and 0.4% type II collagenase (Biosharp, Hefei, China) for 48 h. In particular, after 24 h of digestion, the digestion status of FHN cartilage fragments should be closely monitored to avoid over-digestion. The isolated chondrocytes were resuspended in high-glucose DMEM with 10% fetal bovine serum (FBS, Newzerum, Christchurch, New Zealand) and 1% penicillin-streptomycin. Primary chondrocytes at passages 1 to 3 (P1–P3) were used for subsequent experiments.

### Cell staining

Freshly digested chondrocytes were seeded at a density of 1 × 10^5^ cells per well in 24-well plates (Saining, Suzhou, China) containing sterile coverslips (Servicebio, Wuhan, China). To identify chondrocytes derived from the CON and FHN broilers and evaluate their ECM secretion capacity, cells were cultured for 24 and 48 h. After removing the medium, cells were rinsed with sterile PBS and fixed with 4% PFA for 30 min. Toluidine blue and Alcian blue staining were then performed according to the manufacturer’s instructions. To assess drug-induced ECM changes, chondrocytes were cultured to 70–80% confluence. After medium removal and PBS washing, cells were incubated for 24 h with high-glucose DMEM (Gibco, Wuhan, China) containing fructose-1,6-bisphosphate (FBP, TargetMol, Massachusetts, USA), rotenone (MedChemexpress, New Jersey, USA), or 2-deoxy-D-glucose (2-DG, MedChemexpress, New Jersey, USA), respectively. After treatment, Toluidine blue and Alcian blue staining were performed following the same protocol as described above.

### Cell counting kit-8 (CCK-8) assay

Freshly digested primary chondrocytes were seeded in 96-well plates (Saining, Suzhou, China) at a density of 5,000 cells per well. Primary chondrocytes derived from the CON and FHN groups were cultured for 24, 48, and 72 h to assess the overall proliferative capacity. To determine the optimal treatment concentrations of FBP, rotenone, and 2-DG, chondrocytes were treated under the following conditions: chondrocytes isolated from the CON group were treated for 24 h with FBP at 0, 0.125, 0.25, 0.5, 1, and 2 mM; with rotenone at 0, 0.025, 0.05, 0.1, 0.2, and 0.4 μM; and chondrocytes from the FHN group were treated with 2-DG at 0, 0.01865, 0.0375, 0.075, 0.15, 0.3, 0.6, and 1.2 mM for 24 h. After drug treatment, cell viability was measured using the CCK-8 (Biosharp, Hefei, China) assay according to the manufacturer’s instructions.

### Cellular metabolic activity assay

Primary chondrocytes, freshly digested, were seeded at a density of 5,000 cells per well in 96-well plates. To assess the overall metabolic activity of chondrocytes isolated from the CON and FHN groups, cells were incubated for 24, 48, and 72 h. Subsequently, a 3-[4,5-dimethylthiazole-2-yl]-2,5-diphenyltetrazolium bromide (MTT, Phygene, Fuzhou, China) assay was performed according to the manufacturer’s instructions.

### Extracellular acidification rate and oxygen consumption rate assay

To evaluate mitochondrial OXPHOS and glycolytic activity, extracellular acidification rate (ECAR, Elabscience, Wuhan, China) and oxygen consumption rate (OCR, Elabscience, Wuhan, China) were measured using fluorometric assay kits, according to the manufacturer’s instructions. For OCR measurement, chondrocytes were seeded at a density of 5 × 10^4^ cells per well in black, clear-bottom 96-well plates (Labselect, Guangzhou, China) and cultured until reaching 80-90% confluence. The culture medium was then replaced with 100 μL of the working solution. Prior to measurement, cells were equilibrated in an Infinite M200Pro microplate reader (Tecan, Männedorf, Switzerland) pre-warmed to 37 °C for 30 min. Subsequently, 10 μL of the appropriate drug was added to each well, followed immediately by 50 μL of sealing oil. Fluorescence signals were recorded every 2 min for 120 min at an excitation wavelength of 405 nm and an emission wavelength of 675 nm. For ECAR measurement, cells were seeded at a density of 2 × 10^5^ cells per well in black, clear-bottom 96-well plates and cultured until 80–90% confluence. The culture medium was then replaced with 100 μL of serum- and glucose-free DMEM (Gibco, Wuhan, China) containing the corresponding drug. Plates were equilibrated for 30 min in a microplate reader pre-warmed to 37 °C. Subsequently, 100 μL of working solution was added to each well, and fluorescence signals were recorded every 2 min for 120 min at 37 °C using excitation and emission wavelengths of 490 nm and 535 nm, respectively. ECAR and OCR values were calculated based on the fluorescence signal curves over time. To investigate drug-induced changes in cellular bioenergetics, chondrocytes at 60–70% confluence were treated with FBP, rotenone, or 2-DG, followed by ECAR and OCR measurements as described above.

### ATP assay

Intracellular ATP levels in primary chondrocytes were measured using a luciferase-based bioluminescence assay (Beyotime, Shanghai, China), following the manufacturer’s instructions. The cellular ATP amount was normalized based on cell number (Zhou et al. 2012).

### Extracellular metabolite assay

Freshly digested chondrocytes were seeded at a density of 1 × 10^6^ cells per well in 12-well plates (Saining, Suzhou, China). To investigate extracellular metabolite levels in chondrocytes derived from the CON and FHN broilers, the culture medium was replaced with fresh high-glucose DMEM when cells reached 70–80% confluence. Supernatants were collected at 1, 24, and 48 h after medium replacement, and glucose and lactate (Beyotime, Shanghai, China) concentrations were measured according to the manufacturer’s instructions. To evaluate metabolite changes following drug treatment, primary chondrocytes were cultured to 70–80% confluence. After medium removal and PBS washing, cells were incubated with high-glucose DMEM containing FBP, rotenone, or 2-DG for 24 h. Supernatants were collected, and glucose and lactate concentrations were determined using the same protocol described above.

### Real-time quantitative reverse transcription PCR (qRT-PCR) assays

All primers used in this study are listed in Table 1, and full methodological details are described in the Additional file 1.

**Table 1.** Sequences of primers used to amplify specific mRNAs by qRT-PCR.

Target gene	Primer sequence (5′–3′)	Transcript accession	Product size (bp)
*β-actin*	Forward: AGCGAACGCCCCCAAAGTTCT	NM_205518.2	139
	Reverse: AGCTGGGCTGTTGCCTTCACA		
*Col2a1*	Forward: ACCTACAGCGTCTTGGAGGA	NM_204426.2	155
	Reverse: ATATCCACGCCAAACTCCTG		
*Acan*	Forward: TGCAAGGCAAAGTCTTCTACG	NM_001396161.1	248
	Reverse: GGCAGGGTTCAGGTAAACG		
*Col10a1*	Forward: CAGCTGCCAAATTCAGAATCC	NM_001396427.1	70
	Reverse: GGAAACCTGAGAAAGAAGAATGAACA		
*Mmmp13*	Forward: AGAGACCCTGGAGCACTGATGT	NM_001293090.2	120
	Reverse: GGGATCTCTGTCTCCAGCACCA		
*Adamts5*	Forward: CGTGGTGAAGGTGGTGGTCTTG	XM_040658789.2	106
	Reverse: GTTGTGCTGGTGCTGCCACTT		

### Statistical analysis

Statistical analyses were performed using SPSS software (version 23, IBM, NY, USA). Data are presented as mean ± standard error of the mean (SEM). Normality was assessed prior to statistical testing. For normally distributed data, comparisons between two groups were conducted using an unpaired Student’s t-test, while multiple group comparisons were performed using one-way ANOVA followed by Tukey’s post hoc test. For non-normally distributed data, the Mann–Whitney U test and Kruskal–Wallis test were applied for two-group and multiple-group comparisons, respectively. Differences were considered statistically significant at *p* < 0.05 and highly significant at *p* < 0.01.

## Results

### Functional abnormalities of chondrocytes in FHN broilers

To investigate the pathological changes in articular cartilage and bone associated with FHN in broilers, femurs and tibias were collected from FHS and FHSL broilers for skeletal parameter analysis. As shown in Table 2, both body weight and bone weight (femur and tibia) were significantly lower in FHSL broilers compared with the control group (*p* < 0.01), while no significant difference was observed in bone index (*p* > 0.05). The adverse effects of FHN on bone morphology were primarily manifested in reduced epiphyseal widths. Specifically, the distal epiphysis width of the femur was significantly decreased in both FHS and FHSL broilers compared with the CON group (*p* < 0.05), and the proximal epiphysis width of the tibia was also decreased in FHSL broilers (*p* < 0.05, Table 2). The BMD measurements demonstrated a marked reduction in the total femoral BMD in the FHS and FHSL group compared with the CON group (*p* < 0.01, Table 3). Additionally, the BMD of the proximal femur, distal femur, femoral shaft, tibial shaft, and total tibia also declined significantly in the FHSL groups (*p* < 0.05, Table 3). Biomechanical analysis revealed considerable reductions in the yield force and yield stress of the femur and tibia in both the FHS and FHSL groups (*p* < 0.01, Table 4). Furthermore, FHSL broilers exhibited significant decreases in the tibial ultimate force, ultimate stress, elastic modulus, and stiffness (*p* < 0.05, Table 4).

**Table 2.** Changes in bone morphology parameters of the femur and tibia.

Items	Groups			*p*-value
	CON	FHS	FHSL	
Body weight (kg)	1.24 ± 0.06^a^	1.19 ± 0.08^a^	1.07 ± 0.06^b^	<0.01
Femoral measurements				
Bone weight (g)	7.99 ± 0.17^a^	7.55 ± 0.15^a^	6.75 ± 0.11^b^	<0.01
Bone index	0.64 ± 0.01	0.63 ± 0.01	0.63 ± 0.01	0.498
Length (mm)	71.43 ± 1.94	68.30 ± 0.85	67.68 ± 0.71	0.111
Distal epiphysis width (mm)	20.03 ± 0.59^a^	19.02 ± 0.43^b^	18.86 ± 0.32^b^	0.020
Proximal epiphysis width (mm)	12.62 ± 0.41	12.18 ± 0.51	12.18 ± 0.12	0.655
HED (mm)^1^	8.40 ± 0.19	8.04 ± 0.09	8.10 ± 0.12	0.185
VED (mm)^2^	7.74 ± 0.30	7.34 ± 0.24	7.28 ± 0.08	0.308
Tibial measurements				
Bone weight (g)	10.81 ± 0.25^a^	10.23 ± 0.28^a^	9.17 ± 0.23^b^	<0.01
Bone index	0.87 ± 0.01	0.86 ± 0.02	0.86 ± 0.02	0.773
Length (cm)	92.82 ± 0.73	90.01 ± 1.25	89.49 ± 1.31	0.126
Distal epiphysis width (mm)	18.76 ± 0.42^a^	17.10 ± 0.42^b^	17.30 ± 0.33^b^	0.013
Proximal epiphysis width (mm)	20.92 ± 0.47^a^	19.17 ± 0.45^b^	19.39 ± 0.24^b^	0.011
HED (mm)^1^	7.30 ± 0.18	7.22 ± 0.12	7.19 ± 0.14	0.860
VED (mm)^2^	6.74 ± 0.19	6.34 ± 0.17	6.37 ± 0.11	0.162

Note: *n* = 8, n indicates the number of biologically independent samples per group. Data are all shown as mean ± SEM.

^1^ HED indicates horizontal external diameter.

^2^ VED indicates vertical external diameter.

^a–b^ Means with different superscripts within the same row differ (*p* < 0.05).

**Table 3.** Changes in bone mineral density of the femur and tibia.

Items		Groups			*p-*value
		CON	FHS	FHSL	
Femoral measurements					
BMD^1^ (g/cm^2^)	Total	0.174 ± 0.005^a^	0.154 ± 0.005^b^	0.141 ± 0.005^b^	<0.01
	Proximal femur	0.120 ± 0.003^a^	0.122 ± 0.013^a^	0.083 ± 0.004^b^	0.004
	Femoral shaft	0.261 ± 0.006^a^	0.232 ± 0.010^ab^	0.224 ± 0.009^b^	0.012
	Distal femur	0.092 ± 0.002^a^	0.095 ± 0.012^ab^	0.067 ± 0.003^b^	0.021
BMC^2^ (g)	Total	1.460 ± 0.158	1.216 ± 0.146	1.031 ± 0.058	0.072
	Proximal femur	0.225 ± 0.022^ab^	0.176 ± 0.017^b^	0.140 ± 0.015^bc^	0.012
	Femoral shaft	0.989 ± 0.115	0.804 ± 0.083	0.732 ± 0.045	0.118
	Distal femur	0.247 ± 0.022^a^	0.183 ± 0.014^b^	0.160 ± 0.009^b^	0.003
Tibial measurements					
BMD (g/cm^2^)	Total	0.180 ± 0.003^a^	0.167 ± 0.005^ab^	0.157 ± 0.003^b^	0.002
	Proximal tibia	0.108 ± 0.003	0.131 ± 0.018	0.107 ± 0.003	0.816
	Tibia shaft	0.252 ± 0.007^a^	0.226 ± 0.006^b^	0.211 ± 0.006^b^	0.001
	Distal tibia	0.107 ± 0.003^a^	0.114 ± 0.009^a^	0.092 ± 0.004^b^	0.051
BMC (g)	Total	2.162 ± 0.136^a^	1.896 ± 0.234^ab^	1.656 ± 0.041^b^	0.023
	Proximal tibia	0.344 ± 0.026	0.295 ± 0.029	0.262 ± 0.011	0.066
	Tibia shaft	1.507 ± 0.086^a^	1.262 ± 0.132^ab^	1.156 ± 0.039^b^	0.042
	Distal tibia	0.303 ± 0.025	0.260 ± 0.027	0.239 ± 0.011	0.185

Note: *n* = 8, n indicates the number of biologically independent samples per group. Data are all shown as mean ± SEM.

^1^ BMD indicates bone mineral density.

^2^ BMC indicates bone mineral content.

^a–b^ Means with different superscripts within the same row differ (*p* < 0.05).

**Table 4.** Changes in bone mechanical parameters of the femur and tibia.

Items	Groups			*p* value
	CON	FHS	FHSL	
Femoral measurements				
Stiffness (N/mm)	139.56 ± 7.27	135.28 ± 12.08	120.25 ± 3.11	0.250
Elastic modulus (GPa)	2.92 ± 0.15	2.84 ± 0.25	2.52 ± 0.07	0.250
Yield force (N)	234.15 ± 10.96^a^	193.69 ± 11.95^b^	155.27 ± 6.74^c^	<0.01
Ultimate force (N)	234.81 ± 10.97	195.67 ± 11.74	195.91 ± 15.94	0.073
Yield stress (MPa)	110.42 ± 5.17^a^	91.34 ± 5.64^b^	73.22 ± 3.18^c^	<0.01
Ultimate stress (MPa)	110.73 ± 5.17	92.27 ± 5.54	92.39 ± 7.52	0.073
Tibial measurements				
Stiffness (N/mm)	79.93 ± 2.42^a^	72.73 ± 1.17^ab^	68.04 ± 2.46^b^	0.002
Elastic modulus (GPa)	1.68 ± 0.05^a^	1.52 ± 0.02^ab^	1.43 ± 0.05^b^	0.002
Yield force (N)	188.68 ± 4.43^a^	140.11 ± 10.39^b^	133.13 ± 9.22^b^	<0.01
Ultimate force (N)	203.10 ± 12.61^a^	158.18 ± 8.28^ab^	144.99 ± 8.75^b^	0.003
Yield stress (MPa)	88.98 ± 2.09^a^	66.07 ± 4.90^b^	62.78 ± 4.35^b^	<0.01
Ultimate stress (MPa)	95.77 ± 5.95^a^	74.59 ± 3.90^ab^	68.37 ± 4.13^b^	0.003

Note: *n* = 8, n indicates the number of biologically independent samples per group. Data are all shown as mean ± SEM.

^a–b^ Means with different superscripts within a row differ (*p* < 0.05).

**Figure 1.**
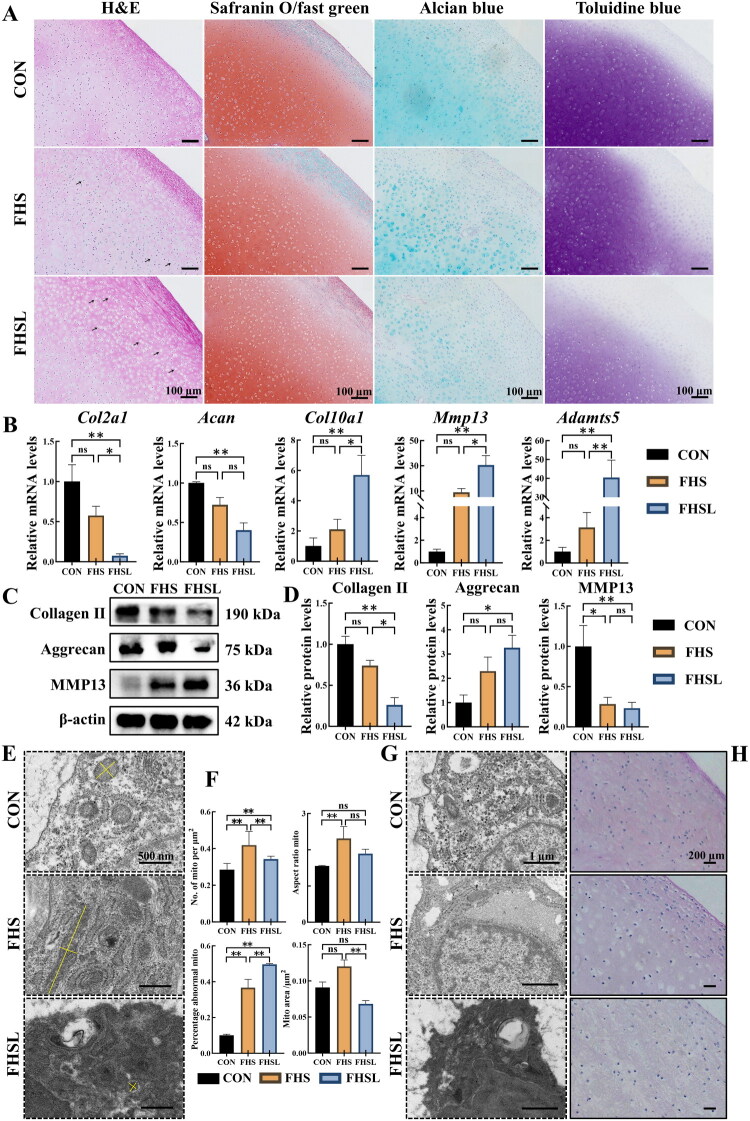
Functional abnormalities of chondrocytes in broilers with femoral head necrosis (FHN). (A) Representative images of hematoxylin and eosin (H&E), Toluidine blue, Alcian blue, and Safranin O/fast Green staining in the femoral head cartilage. The black arrows represent intracytoplasmic vacuolization in chondrocytes. (*n* = 3, scale bar = 100 μm). (B) Relative mRNA expression of *Col2a1*, *acan*, *Col10a1*, *Mmp13*, *Adamts5* in the femoral head cartilage (*n* = 8). (C–D) Western blot analysis of collagen II, aggrecan, and MMP13 in the femoral head cartilage (*n* = 3). (E) Representative transmission electron microscopy (TEM) images of mitochondria in chondrocytes in the femoral head cartilage (*n* = 3, scale bar = 500 nm). The yellow lines represent the schematic measurement of mitochondrial length and width. (F) Quantification of number, aspect ratio, abnormal percentage, and area of mitochondria (mito) in the femoral head cartilage (*n* = 3). (G) Representative TEM images of chondrocytes in the femoral head cartilage (*n* = 3, scale bar = 1 μm). (H) Representative images of periodic acid-schiff (PAS) staining in the femoral head cartilage (*n* = 3, scale bar = 200 μm). *Col2a1,* collagen type II alpha 1; *acan,* aggrecan; *Col10a1,* type Ⅹ collagen alpha 1; *Mmp13,* matrix metalloproteinase 13; *Adamts5,* a disintegrin and metalloproteinase with thrombospondin motifs 5. Beta-actin (β-actin) served as a loading control. Data are all shown as mean ± SEM. ^ns^*P* > 0.05, *p < 0.05, **p < 0.01.

Our previous study demonstrated a strong association between articular cartilage damage and FHN in broilers (Ge et al. 2024). To further elucidate the characteristics of cartilage pathological changes during the progression of FHN. Femoral head cartilage was collected for histological analysis. H&E staining revealed progressively severe intracytoplasmic vacuolization in chondrocytes as FHN progressed (Figure 1(A)). In addition, compared with the CON group, cartilage sections from FHSL broilers showed markedly reduced staining intensity with Safranin O, Toluidine Blue, and Alcian Blue, indicating ECM degradation (Figure 1(A)). qRT-PCR analysis revealed that the expression of matrix synthesis-related genes, including collagen type II alpha 1 (*Col2a1*) and aggrecan (*Acan*), was significantly downregulated in the FHSL group (*p* < 0.01). In contrast, matrix degradation-related genes, including collagen type Ⅹ alpha 1 (*Col10a1*), matrix metalloproteinase 13 (*Mmp13*), and a disintegrin and metalloproteinase with thrombospondin motifs 5 (*Adamts5*), were significantly upregulated (*p* < 0.01, Figure 1(B)). The corresponding protein expression levels were consistent with the mRNA expression trends, further supporting the disruption of ECM homeostasis in FHN-affected broilers (Figures 1(C–D)).

TEM analysis revealed a significant increase in mitochondrial number in chondrocytes from both FHS and FHSL broilers compared with the control group (*p* < 0.01), with a more pronounced elevation observed in the FHS group (*p* < 0.01, Figures 1(E–F)). The mitochondrial aspect ratio was elevated in FHS broilers (*p* < 0.01, Figures 1(E–F)), indicating elongation and a shift toward a fused mitochondrial network. However, as the disease progressed to the FHSL stage, the mitochondrial aspect ratio slightly declined (*p* > 0.05), suggesting enhanced mitochondrial fission and fragmentation (Figures 1(E–F)). Notably, the proportion of morphologically abnormal mitochondria, characterized by swelling, cristae disruption, and vacuolization, increased significantly with FHN progression (*p* < 0.01, Figures 1(E–F)). Further ultrastructural evaluation demonstrated that cytoplasmic glycogen granules in FHSL chondrocytes were markedly decreased compared with the CON group (Figure 1(G)). Consistently, an attenuation in PAS staining intensity was observed in the cartilage of FHSL broilers (Figure 1(H)).

### Metabolic characteristics of cartilage in FHN broilers

**Figure 2.**
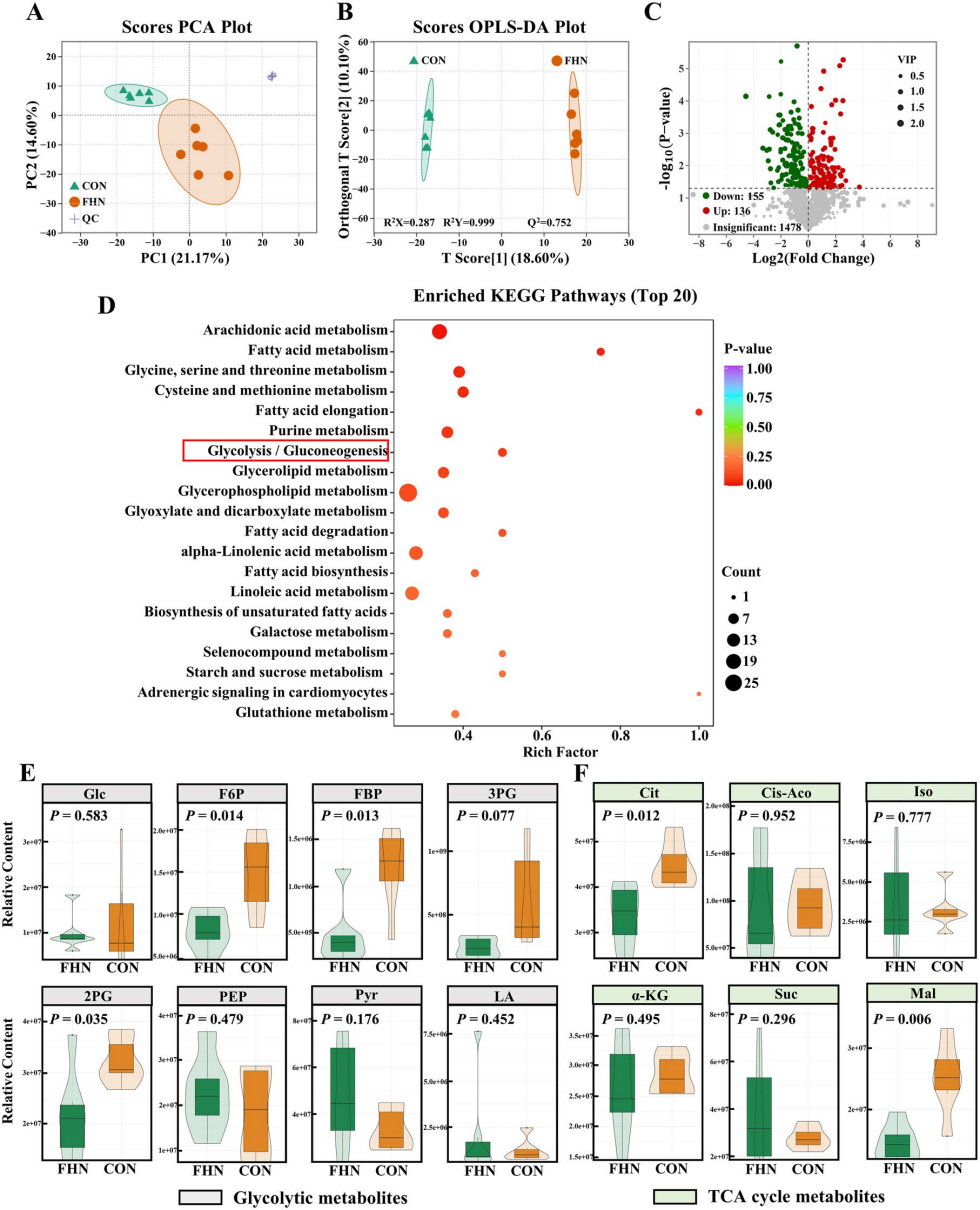
Non-targeted metabolome profiling of femoral head cartilage in FHN and control (CON) broilers. (A) Principal component analysis (PCA) score plot based on non-targeted metabolomic profiles of femoral head cartilage (*n* = 6). (B) Orthogonal partial least squares discriminant analysis (OPLS-DA) score plot based on non-targeted metabolomic profiles of femoral head cartilage (*n* = 6). (C) Volcano plot of differentially expressed metabolites (DEMs) based on non-targeted metabolomic profiles of femoral head cartilage in the CON and FHN groups (*n* = 6). (D) 20 significantly enriched metabolic pathways based on non-targeted metabolomic profiles of femoral head cartilage in the CON and FHN groups (*n* = 6). (E) Violin plots show the relative content of glycolytic metabolites based on the non-targeted metabolome of femoral head cartilage in the CON and FHN groups (*n* = 6). (F) Violin plots show the relative content of the tricarboxylic acid (TCA) cycle metabolites based on the non-targeted metabolome of femoral head cartilage in the CON and FHN groups (*n* = 6). Glc: glucose; F6P: fructose-6-phosphate; FBP: fructose-1,6-bisphosphate; 3PG: 3-phosphoglycerate; 2PG: 2-phosphoglycerate; PEP: phosphoenolpyruvate; pyr: pyruvate; LA: lactate; cit: citrate; Cis-Aco: cis-aconitate; iso: isocitrate; α-KG: α-ketoglutarate; suc: succinate; mal: malate.

The coexistence of mitochondrial dysfunction and glycogen reduction in FHN chondrocytes prompted further investigation into their metabolic characteristics to assess whether these alterations serve as potential drivers of cartilage homeostasis disruption. Non-targeted metabolomic profiling of femoral head cartilage from FHN and CON broilers was performed using LC-MS/MS. Unsupervised PCA analysis revealed a distinct separation in metabolite profiles between the FHN and CON groups (Figure 2(A)), indicating substantial metabolic differences. This separation was further supported by supervised OPLS-DA, which demonstrated excellent model fit and predictive accuracy (Figure 2(B), R^2^Y = 0.999, Q^2^ = 0.752). As illustrated in the volcano plot, a total of 291 DEMs were identified, comprising 136 upregulated and 155 downregulated metabolites (Figure 2(C)). The top 20 significantly enriched KEGG pathways highlighted associations with carbohydrate metabolism and lipid metabolism, such as glycolysis/gluconeogenesis, starch and sucrose metabolism, galactose metabolism, fatty acid metabolism, and fatty acid biosynthesis (Figure 2(D)). Considering that chondrocytes rely predominantly on glycolysis for energy production (Liao et al. 2024), the alterations in the glycolysis/gluconeogenesis pathway were of particular interest. Compared with the CON group, the glycolytic intermediates fructose-6-phosphate (F6P), fructose-1,6-bisphosphate (FBP), and 2-phosphoglycerate (2PG) were significantly diminished in FHN cartilage (*p* < 0.05), while the terminal product pyruvate (Pyr) showed an upward trend (*p* > 0.05, Figure 2(E)). Furthermore, citrate (Cit) and malate (Mal), representing the early- and late-stage intermediates of the tricarboxylic acid (TCA) cycle, respectively, were markedly reduced in the FHN group (*p* < 0.05, Figure 2(F)), suggesting a potential suppression of OXPHOS.

**Figure 3.**
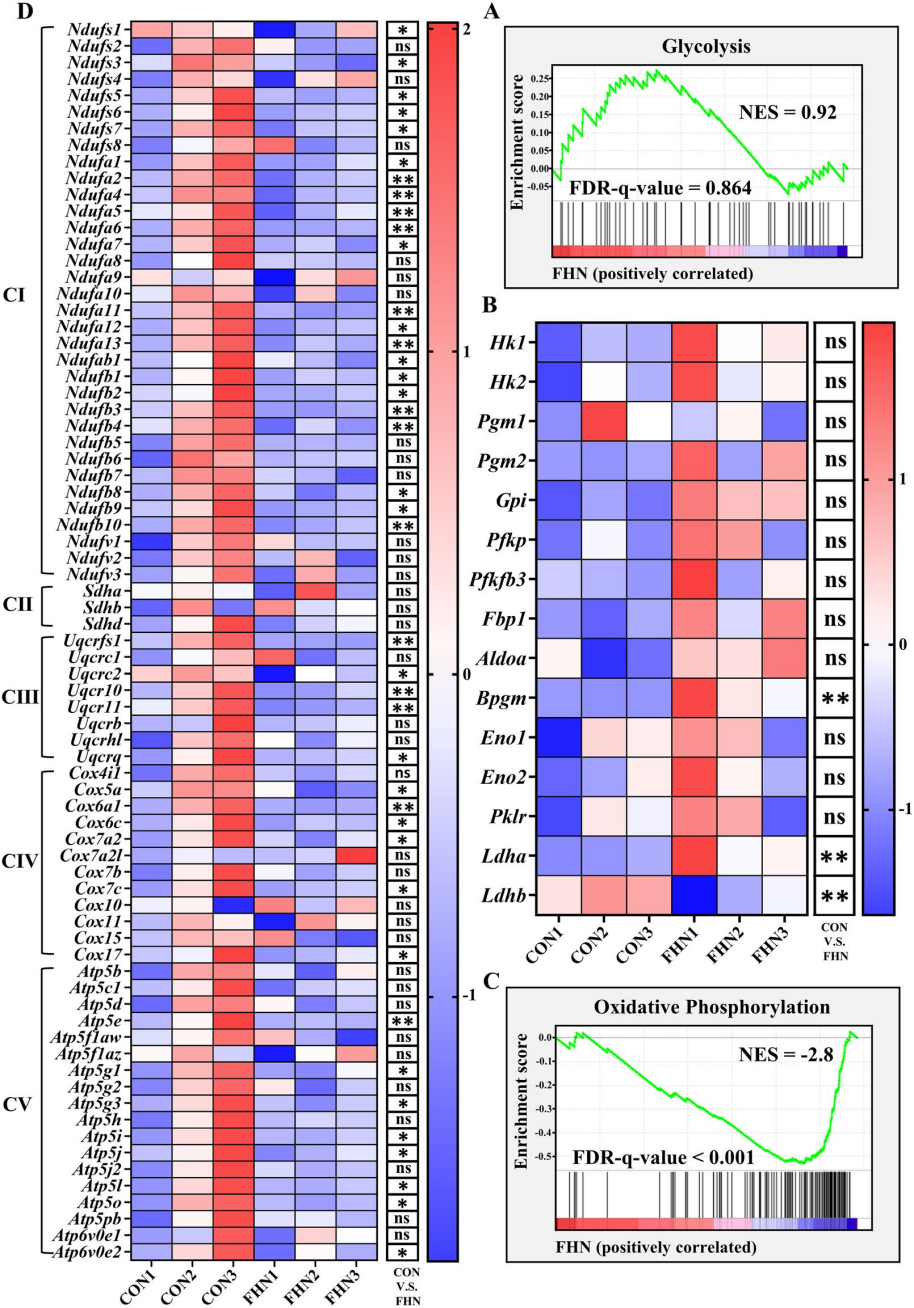
RNA sequencing (RNA-seq) analysis of femoral head cartilage in FHN and CON broilers. (A) The gene set enrichment analysis (GSEA) plot shows enrichment of glycolysis gene expression scores based on the RNA-seq analysis of femoral head cartilage in the CON and FHN groups (*n* = 3). (B) Heatmap of the relative expression of selected glycolysis-related genes based on the RNA-seq results of femoral head cartilage in the CON and FHN groups (*n* = 3). The RNA-seq results were normalized using fragments per kilobase of transcript per million mapped reads (FPKM) and log_2_-transformed. (C) The GSEA plot shows enrichment of oxidative phosphorylation gene expression scores based on the RNA-seq analysis of femoral head cartilage in the CON and FHN groups (*n* = 3). (D) Heatmap of the relative expression of selected genes encoding mitochondrial complexes based on the RNA-seq results of femoral head cartilage in the CON and FHN groups (*n* = 3). The RNA-seq results were normalized using fragments per kilobase of transcript per million mapped reads (FPKM) and log_2_-transformed. NES: normalized enrichment score; CI: complex I; CII: complex II; CIII: complex III; CIV: complex IV; CⅤ: complex Ⅴ.

To further elucidate the metabolic alterations between glycolysis and OXPHOS in FHN cartilage, GSEA based on transcriptomic data were conducted. The results showed that the expression of glycolysis pathway-related genes in FHN cartilage was overall similar to that in CON cartilage, but exhibited a positive enrichment tendency (NES = 0.92, Figure 3(A)). Notably, the significant upregulation of lactate dehydrogenase A (*Ldha*) and bisphosphoglycerate mutase (*Bpgm*), with the downregulation of lactate dehydrogenase A (*Ldhb*) in FHN cartilage (*p* < 0.01, Figure 3(B)) further indicated aberrant activation of glycolytic metabolism. In contrast, GSEA revealed significant downregulation of OXPHOS-related genes in FHN cartilage (NES = −2.8, Figure 3(C)). Heatmap analysis further demonstrated the widespread suppression of the expression of genes encoding structural subunits and assembly factors of mitochondrial respiratory complexes I to V (Figure 3(D)). These findings suggest that glycolytic reprogramming, characterized by enhanced glycolysis and impaired OXPHOS, occurs in FHN cartilage.

### Glycolytic reprogramming in chondrocytes from FHN broilers

**Figure 4.**
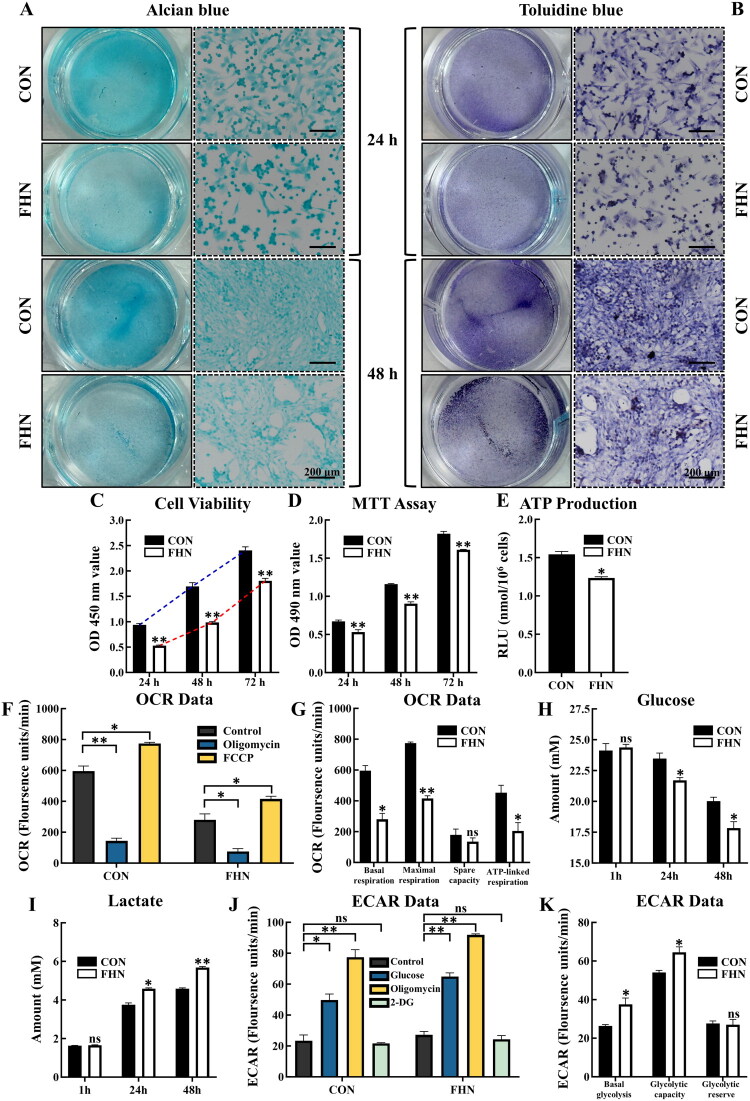
Glycolytic reprogramming in chondrocytes from FHN broilers. (A–B) Alcian blue and toluidine blue staining of primary chondrocytes from CON and FHN broilers after culturing for 24 and 48 h (*n* = 3). Left images display the overall view of representative well (24-well plate) in alcian blue and toluidine blue staining, and right images display microscopic images obtained from the corresponding wells (scale bar = 200 μm). (C) Cell viability of primary chondrocytes from CON and FHN broilers after culturing for 24, 48, and 72 h (*n* = 3). The blue and red dotted lines represent the connecting lines of the average optical density (OD) values of chondrocytes in the CON group and FHN group after culturing for 24, 48, and 72 h, respectively. (D) MTT assay of chondrocytes in the CON group and FHN group after culturing for 24, 48, and 72 h (*n* = 3). (E) ATP levels of chondrocytes in the CON group and FHN group (*n* = 3). (F–G) Oxygen consumption rate (OCR) data of chondrocytes in the CON group and FHN group (*n* = 3). (H) Content of glucose in culture medium at 1, 24, and 48 h (*n* = 3). (I) Content of lactate in culture medium at 1, 24, and 48 h (*n* = 3). (J–K) extracellular acidification rate (ECAR) data of chondrocytes in the CON group and FHN group (*n* = 3). Data are all shown as mean ± SEM. ^ns^*P* > 0.05, *p < 0.05, **p < 0.01.

To verify whether the metabolic characteristics observed in cartilage tissue could be recapitulated at the cellular level, primary chondrocytes were isolated from the femoral head cartilage of FHN and CON broilers for subsequent functional analysis. After 24 and 48 h of culture, Alcian blue and Toluidine blue staining revealed reduced staining intensity in FHN chondrocytes relative to CON chondrocytes (Figures 4(A–B)). During the 72-h culture period, both FHN and CON chondrocytes remained in the logarithmic growth phase, and the viability of FHN chondrocytes was significantly lower at 24, 48, and 72 h (*p* < 0.01, Figure 4(C)). Mitochondrial oxidoreductase activity, as assessed by the MTT assay, was markedly decreased in FHN chondrocytes compared with CON chondrocytes (*p* < 0.01, Figure 4(D)). Moreover, intracellular ATP levels were significantly reduced in the FHN group (*p* < 0.05, Figure 4(E)). Consistent with these results, the OCR assay revealed significantly lower basal respiration, maximal respiration, and ATP-linked respiration in FHN chondrocytes (*p* < 0.05, Figures 4(F–G)). Furthermore, glucose consumption and lactate production were significantly elevated in FHN chondrocytes at both 24 and 48 h (*p* < 0.05, Figures 4(H–I)). ECAR assay revealed enhanced basal glycolysis and glycolytic capacity in FHN chondrocytes compared with CON chondrocytes (*p* < 0.05, Figures 4(J–K)). Collectively, these results suggest that glycolytic reprogramming is present in FHN chondrocytes, aligning with the metabolic alterations previously identified at the tissue level.

### Glycolytic reprogramming impairs chondrocyte function

**Figure 5.**
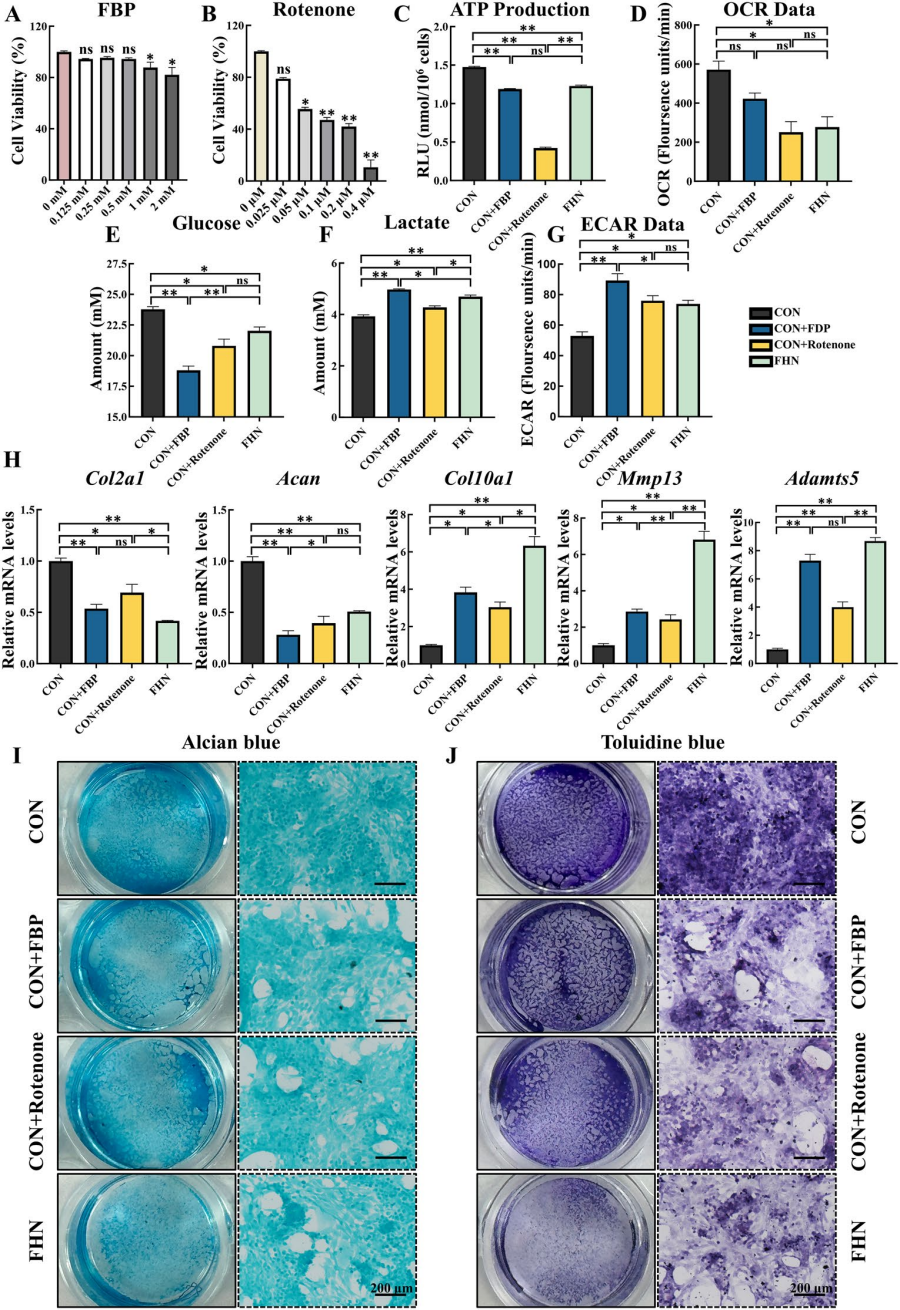
Glycolytic reprogramming impairs chondrocyte function. (A) Cell viability of CON chondrocytes after treatment with 0, 0.125, 0.25, 0.5, 1, 2 mM FBP for 24 h (*n* = 3). (B) Cell viability of CON chondrocytes after treatment with 0, 0.025, 0.05, 0.1, 0.2, 0.4 μM rotenone for 24 h (*n* = 3). (C) ATP levels of CON chondrocytes with treatment of FBP or rotenone for 24 h, CON and FHN chondrocytes as controls (*n* = 3). (D) OCR data of CON chondrocytes with treatment of FBP or rotenone for 24 h, with untreated CON and FHN chondrocytes as controls (*n* = 3). (E–F) Content of glucose and lactate in culture medium of CON chondrocytes with treatment of FBP or rotenone for 24 h, with untreated CON and FHN chondrocytes as controls (*n* = 3). (G) ECAR data of CON chondrocytes with treatment of FBP or rotenone for 24 h, with untreated CON and FHN chondrocytes as controls (*n* = 3). (H) Relative mRNA expression of *Col2a1*, *acan*, *Col10a1*, *Mmp13*, and *Adamts5* of CON chondrocytes with treatment of FBP or rotenone for 24 h, with untreated CON and FHN chondrocytes as controls (*n* = 3). (I–J) alcian blue and toluidine blue staining of CON chondrocytes with treatment of FBP or rotenone for 24 h, with untreated CON and FHN chondrocytes as controls (*n* = 3). Left images display the overall view of representative wells (24-well plate) in alcian blue and toluidine blue staining, and right images display microscopic images obtained from the corresponding wells (scale bar = 200 μm). Data are all shown as mean ± SEM. ^ns^*P* > 0.05, *p < 0.05, **p < 0.01.

Exogenous supplementation with either FBP (key glycolytic intermediate) or rotenone (mitochondrial complex I inhibitor) has been reported to enhance glycolytic flux while suppressing OXPHOS (Durairaj et al. 2009; Zhang et al. 2024; Li et al. 2025; Miao et al. 2022; Cui et al. 2024). To investigate the potential impact of glycolytic reprogramming on chondrocyte function, primary chondrocytes from CON broilers were treated with FBP or rotenone to mimic the metabolic alterations observed in FHN chondrocytes. Based on CCK-8 assays, no significant cytotoxicity was detected following treatment with 1 mM FBP or 0.025 μM rotenone in CON chondrocytes (Figures 5(A–B)), and these doses were, therefore, selected for subsequent experiments. FBP treatment markedly reduced ATP production in CON chondrocytes (*p* < 0.01), reaching levels comparable to those in the FHN group (*p* > 0.05, Figure 5(C)). Rotenone decreased both ATP production and OCR (*p* < 0.05) in CON chondrocytes, with ATP levels reduced below, (*p* < 0.05) and OCR reduced to those (*p* > 0.05) in FHN chondrocytes (Figures 5(C–D)). Additionally, treatment with FBP or rotenone significantly elevated ECAR (*p* < 0.05, Figure 5(G)), along with increased glucose consumption and lactate production (*p* < 0.05, Figures 5(E–F)) in CON chondrocytes, glucose and ECAR reaching levels comparable to those in the FHN group. qRT-PCR analysis demonstrated a significant downregulation of the expression of matrix synthesis-related genes and an upregulation of genes associated with matrix degradation in CON chondrocytes treated with FBP or rotenone (*p* < 0.05, Figure 5(H)). Toluidine blue and Alcian blue staining revealed a reduction in staining intensity in CON chondrocytes following FBP or rotenone treatment (Figures 5(I–J)). These findings collectively indicate that induced glycolytic reprogramming impairs matrix homeostasis in chondrocytes.

### Glycolysis suppression restores FHN chondrocyte function

**Figure 6.**
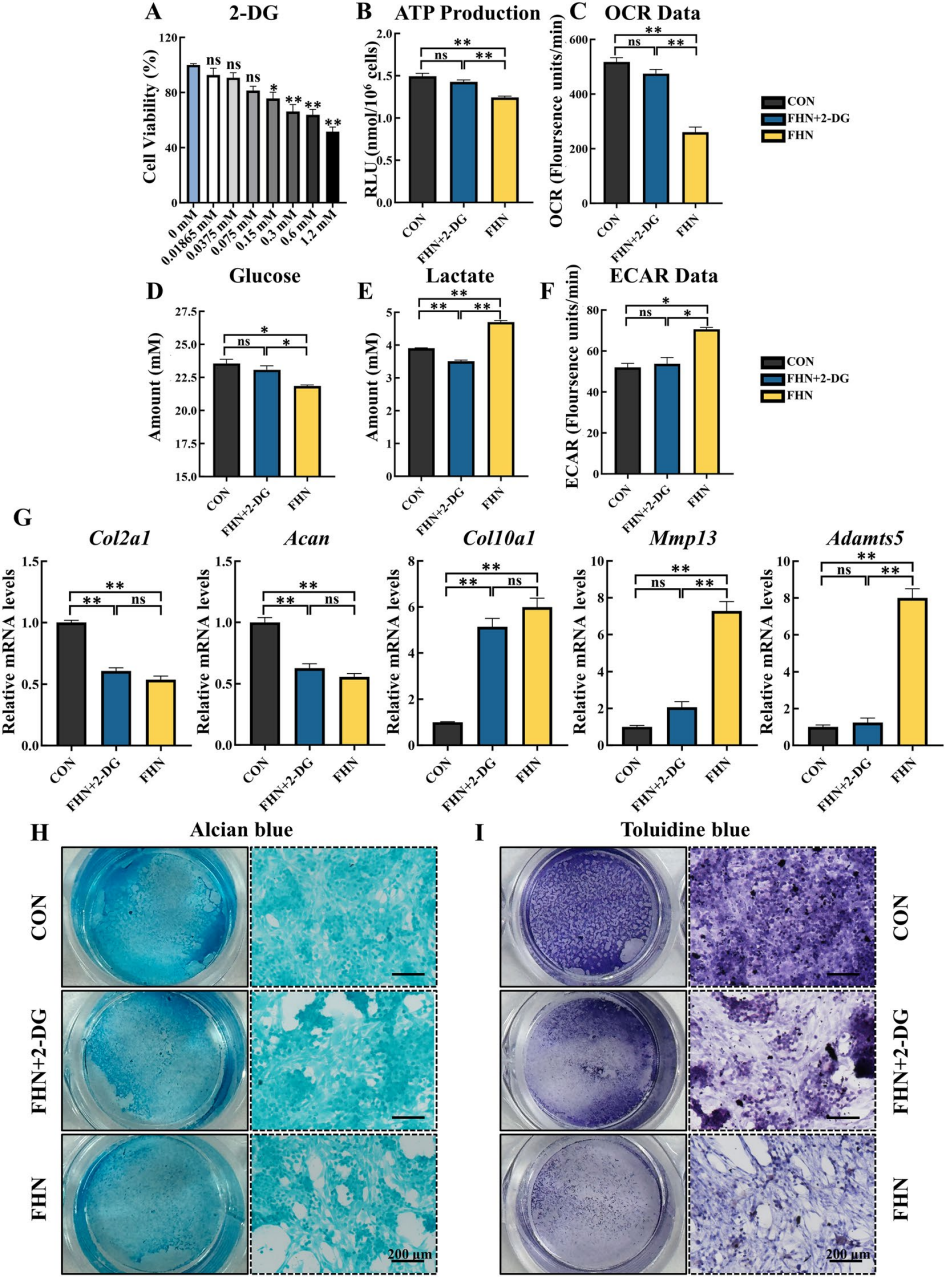
Glycolysis suppression restores FHN chondrocyte function. (A) Cell viability of FHN chondrocytes after treatment with 0, 0.01865, 0.0375, 0.075, 0.15, 0.3, 0.6, 1.2 mM 2-deoxy-D-glucose (2-DG) for 24 h (*n* = 3). (B) ATP levels of FHN chondrocytes with 2-DG treatment for 24 h, with untreated CON and FHN chondrocytes as controls (*n* = 3). (C) OCR data of FHN chondrocytes with 2-DG treatment for 24 h, with untreated CON and FHN chondrocytes as controls (*n* = 3). (D–E) Content of glucose and lactate in culture medium of FHN chondrocytes with 2-DG treatment for 24 h, with untreated CON and FHN chondrocytes as controls (*n* = 3). (F) ECAR data of FHN chondrocytes with 2-DG treatment for 24 h, with untreated CON and FHN chondrocytes as controls (*n* = 3). (G) Relative mRNA expression of *Col2a1*, *acan*, *Col10a1*, *Mmp13*, and *Adamts5* of FHN chondrocytes with 2-DG treatment for 24 h, with untreated CON and FHN chondrocytes as controls (*n* = 3). (H–I) alcian blue and toluidine blue staining of FHN chondrocytes with 2-DG treatment for 24 h, with untreated CON and FHN chondrocytes as controls (*n* = 3). Left images display the overall view of representative wells (24-well plate) in alcian blue and toluidine blue staining, and right images display microscopic images obtained from the corresponding wells (scale bar = 200 μm). Data are all shown as mean ± SEM. ^ns^*P* > 0.05, *p < 0.05, **p < 0.01.

To determine whether the impaired function of FHN chondrocytes could be restored by regulating glycolysis, cells were treated with 2-DG, a classical glycolysis inhibitor. CCK-8 assays indicated no significant cytotoxicity following treatment with 0.15 mM 2-DG in FHN chondrocytes (Figure 6(A)), and this dose was subsequently applied. 2-DG elevated ATP production and OCR in FHN chondrocytes (*p* < 0.05), restoring both to the level of CON chondrocytes (*p* > 0.05, Figures 6(B–C)). Furthermore, treatment with 2-DG significantly suppressed glucose consumption (*p* < 0.05, Figure 6(D)), lactate production (*p* < 0.05, Figure 6(E)), and ECAR (*p* < 0.05, Figure 6(F)), and glucose and ECAR levels returned to control levels (*p* > 0.05), while lactate levels reduced below than control levels (*p* < 0.05). Although the expression of matrix synthesis-related genes was not significantly upregulated in FHN chondrocytes under 2-DG treatment (*p* > 0.05), the expression of matrix degradation-related genes was notably downregulated (*p* < 0.05) to a level comparable to that of the CON chondrocytes (*p* > 0.05, Figure 6(G)). Toluidine blue and Alcian blue staining also demonstrated enhanced staining intensity in FHN chondrocytes following 2-DG treatment (Figures 6(H–I)). Together, these results indicate that suppression of aberrant glycolysis *via* 2-DG decreases catabolic activity in FHN chondrocytes.

## Discussion

FHN is the major leg disorder in intensive broiler farming, and its pathological features and potential mechanisms are the focus of investigations. In this study, skeletal parameters, including bone mineral density, mechanical properties, and bone morphology, were negatively impacted to varying degrees as FHN progressed. In addition, matrix metabolic disorder and ECM degradation were observed in FHN cartilage. These results are consistent with our previous findings, further supporting the characterization of progressive bone quality decline and cartilage degeneration during FHN development (Ge et al. 2024). Abnormal mechanical stress on the hip joint is a significant etiology in FHN (Tan et al. 2021; Elgaz et al. 2020). Given that cartilage serves as the cushioning and lubricating structure within the hip joint, its pathological alterations require further investigation.

Numerous studies indicated that ECM alterations in cartilage were associated with mitochondrial dysfunction (Guo et al. 2025; Xiao et al. 2024). Ultrastructural analysis revealed a tendency shift in mitochondrial morphology toward a fused mitochondrial network in FHS chondrocytes, whereas smaller mitochondria from mitochondrial fission were evident in FHSL chondrocytes. Mitochondria are dynamic organelles that maintain their quality through continuous fusion and fission, collectively referred to as mitochondrial dynamics (Grel et al. 2023). These findings suggest abnormal mitochondrial dynamics during the development of FHN. Mitochondrial elongation *via* fusion preserves mitochondrial DNA (mtDNA) integrity to sustain OXPHOS efficiency (Grel et al. 2023; Santel et al. 2003). In contrast, excessive fission could disrupt the electron transport chain (ETC) and promote a shift toward glycolysis (Ansari et al. 2022; Liesa and Shirihai 2013). Therefore, the mitochondrial fusion in FHS chondrocytes may reflect an adaptive response to restore mitochondrial function to meet the high energy required during early disease compensation. Furthermore, the presence of smaller mitochondria in FHSL chondrocytes suggested mitochondrial dysfunction. Disruption of mitochondrial dynamics may disturb the initial balance between OXPHOS and glycolysis in chondrocytes (Buck et al. 2016). Chondrocytes primarily rely on glycolysis to utilize glucose (Wu et al. 2022). Besides being the substrate for glycolysis, glucose could be stored as glycogen in the cytoplasm through the synergistic effect of hexokinase, phosphoglucomutase, and glycogen synthase, providing a short-term energy reserve (Gentry et al. 2022; Wang et al. 2021). In this study, glycogen granules were significantly reduced in FHSL chondrocytes compared with CON chondrocytes, consistent with findings by Wang et al. in osteoarthritis (OA) chondrocytes (Wang et al. 2023). Rapid mobilisation of glycogen stored in chondrocytes may be to sustain glycolytic ATP production for energy deficits caused by mitochondrial dysfunction during FHN progression.

Non-targeted metabolomic and transcriptomic profiling demonstrated significant enrichment of the glycolytic pathway in FHN cartilage, accompanied by a positive enrichment trend of glycolysis-related genes and significant downregulation of OXPHOS-related genes. These results suggest that FHN chondrocytes undergo glycolytic reprogramming, resembling the metabolic alterations previously reported in OA cartilage (Henry and O’Neill 2025). OCR and ECAR are considered the gold standards for assessing OXPHOS and glycolysis, respectively (Chen et al. 2025). In FHN chondrocytes, elevated basal ECAR and decreased basal OCR further support a metabolic shift toward glycolysis and impaired mitochondrial respiration. Notably, approximately 75% of the top 20 enriched KEGG pathways were related to carbohydrate, lipid, and amino acid metabolism. Since mitochondria serve as the core of these fundamental metabolic processes (Hong et al. 2022), the enrichment feature reflects widespread mitochondrial dysfunction in FHN chondrocytes. Moreover, the glutathione metabolism pathway, which is critically involved in antioxidant defense (Aquilano et al. 2014), was also significantly enriched, implying elevated oxidative stress in FHN chondrocytes. ROS is mainly produced in mitochondria and this finding also suggests the presence of mitochondrial dysfunction in FHN chondrocytes.

TCA cycle utilizes acetyl-Coenzyme A, which is converted from pyruvate, as a metabolic substrate and produces nicotinamide adenine dinucleotide (NADH) and reduced flavin adenine dinucleotide (FADH_2_) through a series of enzymatic reactions, both of which are the key electron donors for the ETC (Nsiah-Sefaa and McKenzie 2016). In this study, TCA cycle intermediates, Cit and Mal, were significantly reduced in FHN cartilage, indicating the TCA cycle was inhibited. The suppression of the TCA cycle weakened OXPHOS levels, leading to oxidative stress damage (Schenkl et al. 2023). Glycolytic intermediates, including F6P, FBP, and 2PG, were rapidly consumed, combined with high expression of *Ldha* in FHN cartilage and high lactate production in FHN chondrocytes *in vitro*, suggesting that pyruvate was diverted away from the TCA cycle, and predominantly metabolized through lactate fermentation. Together, these findings suggest that FHN chondrocytes increase glycolytic flux at the expense of OXPHOS (Jackson et al. 2017). Interestingly, a considerable upregulation of *Bpgm* gene was first observed in FHN cartilage. BPGM is primarily known for its ability to maintain oxygen homeostasis by regulating the oxygen-binding capacity of hemoglobin (Huang et al. 2024; Cho et al. 2008), yet it has been rarely reported in cartilage. Zhang et al. found that the hemoglobin body (Hedy) exists in chondrocytes to adapt to hypoxic environments (Zhang et al. 2023). Whether the upregulation of the *Bpgm* gene is associated with Hedy or with hypoxia exacerbated by mitochondrial dysfunction in FHN cartilage remains to be further investigated.

Under abnormal conditions, glycolytic reprogramming is considered an adaptive mechanism in chondrocytes to maintain energy homeostasis and promote cell survival (Zheng et al. 2021; Pi et al. 2024). However, excessive activation of glycolysis may have adverse effects on chondrocyte activity and function. In this study, FBP or rotenone treatment enhanced glycolysis and suppressed OXPHOS in CON chondrocytes, effectively recapitulating the metabolic characteristics of FHN chondrocytes. Metabolic shifts in CON chondrocytes led to matrix metabolic disruption and ECM degradation, resembling the pathological features of FHN cartilage. 2-DG, a glucose analog that competitively inhibits hexokinase, is widely used as a glycolysis inhibitor (Wu et al. 2023; O’Neill et al. 2019). In FHN chondrocytes, 2-DG treatment reduced lactate production and downregulated the expression of matrix degradation-related genes, indicating that targeting glycolysis can partially restore the function of FHN chondrocytes and alleviate EXM homeostasis imbalance. In addition, 2-DG treatment enhanced ATP production and mitochondrial respiration in FHN chondrocytes, aligning with recent findings by Aiestaran-Zelaia et al. that 2-DG enhances mitochondrial function in the heart (Aiestaran-Zelaia et al. 2022). These results suggest that 2-DG exerts pleiotropic effects on cellular metabolism. Lactate was initially considered a metabolic waste of anaerobic glycolysis. However, growing evidence has identified it as a crucial signaling molecule with broad regulatory functions (Brooks 2018). Exogenous lactate increased the expression of *Mmp13*, *Mmp3*, and *Adamts4* while inhibiting *Col2a1* expression in chondrocytes (Huang et al. 2023). Similarly, a lactate-induced extracellular acidified environment impaired ECM homeostasis in cartilage (Zhang et al. 2016). Moreover, LDH promoted ROS generation by interacting with NADH, contributing to ECM degradation in OA (Arra et al. 2020). Therefore, ECM destruction in cartilage may be closely associated with lactate signaling activated by glycolytic reprogramming during the development of FHN in broilers.

## Conclusion

**Figure 7.**
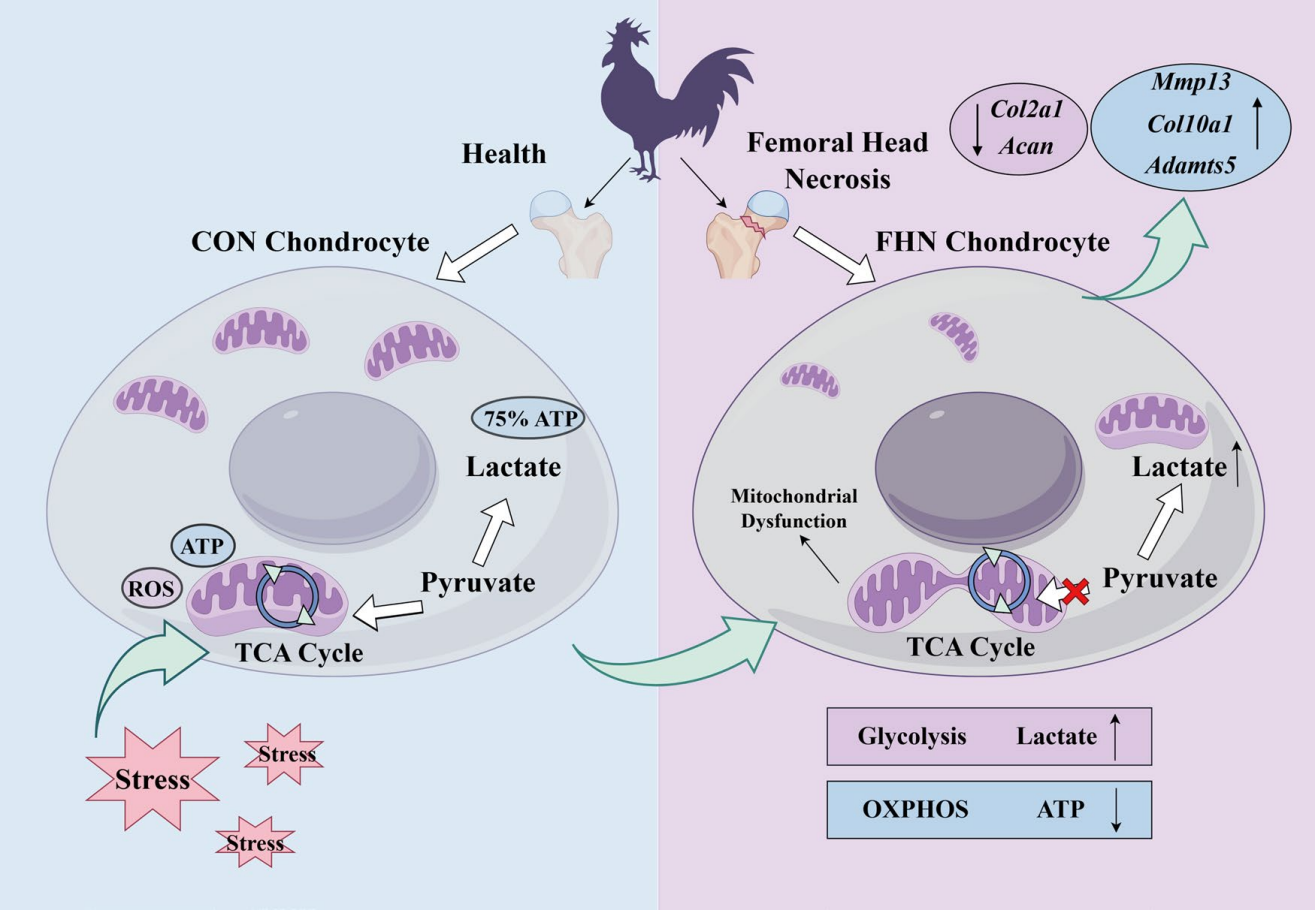
Glycolytic reprogramming impairs the ECM homeostasis in femoral head cartilage of FHN broilers.

In conclusion, this study systematically revealed the metabolic features of femoral head cartilage in FHN broilers for the first time. The results indicate that FHN chondrocytes undergo glycolytic reprogramming, characterized by enhanced glycolytic flux and suppressed OXPHOS. This unique metabolic shift was consistently confirmed at both the tissue and cellular levels. Importantly, simulation of glycolytic reprogramming in healthy chondrocytes induced cartilage ECM destruction resembling that observed in FHN, while inhibition of glycolysis by 2-DG downregulated the expression of matrix degradation-related genes and partially restored mitochondrial function, suggesting that glycolytic reprogramming may lead to the imbalanced ECM homeostasis of cartilage in FHN broilers (Figure 7). These findings provide a new research perspective for elucidating the pathological mechanisms of FHN in broilers and a theoretical foundation for screening feed additives that prevent FHN in broilers.

## Supplementary Material

Additional file 1.docx

## Data Availability

The RNA-seq data had been submitted to the SRA database with the accession number PRJNA1211264. Other datasets used during the current study are available from the corresponding author on reasonable request.
