# Supplementary material for: Glycolytic reprogramming impairs chondrocyte function in broilers with femoral head necrosis
Source: Vet Q. 2025 Oct 29;45(1):2579940. doi: 10.1080/01652176.2025.2579940 (PMC12573555; doi:10.1080/01652176.2025.2579940)
Supplement: Additional file 1.docx [file TVEQ_A_2579940_SM8663.docx]

**Western Blot Analysis**

Total protein was extracted from cartilage tissue using radio immunoprecipitation assay lysis buffer (RIPA, Beyotime, Shanghai, China) supplemented with protease inhibitors (Servicebio, Wuhan, China) and quantified using a BCA Protein Assay Kit (Beyotime, Shanghai, China). Equal protein loads were resolved using sodium dodecyl sulfate-polyacrylamide gel electrophoresis (SDS-PAGE) and transferred onto polyvinylidene difluoride (PVDF, Merck Millipore, Darmstadt, Germany) membranes. Menbranes were blocked for 2 h at room temperature and subsequently incubated overnight at 4 °C with primary antibodies for target proteins. Following three washes with Tris-buffered saline containing 0.1% Tween-20 (TBST, Servicebio, Wuhan, China), the membranes were incubated with horseradish peroxidase (HRP)-conjugated anti-rabbit secondary antibodies (Abcam, Cambridge, UK) at room temperature for 2 h. Protein bands were visualized using enhanced chemiluminescence (ECL, Beyotime, Shanghai, China) reagents, and images were captured using a gel imaging system (Bio-Rad, Hercules, CA, USA). Band intensities were analyzed with using ImageJ software (NIH, Bethesda, MD, USA). The primary antibodies used included anti-collagen Ⅱ (1:500, Proteintech, Wuhan, China), anti-aggrecan (1:500, Proteintech, Wuhan, China), anti-MMP13 (1:500, Proteintech, Wuhan, China), and anti-β-actin (1:300, Servicebio, Wuhan, China).

**Real-Time Quantitative Reverse Transcription PCR (qRT-PCR) assays**

Total RNA was extracted from cartilage tissue and chondrocytes using TRIzol reagent (Angel Gene, Nanjing, China). According to the manufacturer’s instructions, reverse transcription was carried out with the HiScript Ⅲ All-in-one RT SuperMix (Vazyme, Nanjing, China), and qRT-PCR was conducted using the ChamQ Blue Universal SYBR qPCR Master Mix (Vazyme, Nanjing, China) on a 7300 Real-Time PCR System (Applied Biosystems, California, USA). Relative mRNA expression levels were calculated using the 2^–ΔΔCt^ method. All primers used in this study are listed in Table 1.
